# Supplementary material for: Chromothripsis during telomere crisis is independent of NHEJ, and consistent with a replicative origin
Source: Genome Res. 2019 May;29(5):737–49. doi: 10.1101/gr.240705.118 (PMC6499312; doi:10.1101/gr.240705.118)
Supplement: Supplemental Material [file supp_gr.240705.118_Supplemental_file_1.zip › contigs/annotated_contigs/DB54/contig.4.DB54_length_1204_mean_cov_9.2134551495.docx]

**DB54_length_1204_mean_cov_9.2134551495**

```
TATTTCTGTTAAATGTCTTGATGCCTAGTTAAATTATTTGACGACCCTTTTGCTCCTGTCAAGCCTGGGCCTTTGTTAGTTTGTGCTTA
 >chrUn_gl000241:29730-30343 - E=1e-285 p=4e-02
TTTATTAGATTTTTGCCTGCAGACTTAGACAGTGACTCTTACTCTAGGAAAGATTCATCCTCATGGGCCTCAGCCACATGTTCTAGGTA

TACTTTGTGAGTTCTCTCCACTCTGCTATGTCCCAAATTTGTGTGATCTCTGGCATCTCCACTCAGCCCTCAGAAGTGCCAGCCACTCT

GCAGAGGCCTTGTGGAGCCTGCCTACTGTATGCACTCCCCCAGCCCTTGGCCCCAGACCTGCAGAGAACTTTTGCATACTCTTTTGAGG

CCTCACCTGTATGTAGTTCCCTCTTCTCCAGTACCTTATTCTATAAACTCCAAACACCTTAGCACTGCAAGACTCTCAGCTTAGAGAAA

GTGGCATTGCCTCATTTTTGGACGTCTCTACCTCTCTCTGCATGGTCAAGAAACTGCCATTGGGCAGAAAACAGAAGTATGTGTGAGAT

TTGCCTCCTGTGTTTTCCTGTTCTCAAATATCACAGTCCTGTTCTGCCTGTGTTCCAATCCCTGGAAACAGTTCTCTC|T|AAAAGAGC
                                                                                >chr1:1
TCTGATAATCTTGTTAGGCTGAATTTAGACTGTTACAGAATGTCTTTTTTTCTTTTTTACCTCTTCTACTTGTAAATGCATTTATGAGT
93315685-193315824 + E=1e-71 p=1e-03
TCTTTCTGAATTTTGAGTATCTGTGTTTCACCTGAAAGCCT|G|TTAAGCTGTAATTGGTACTATAGGTACACCAGTAATTTTTTTTTT
                                          >chr1:193314890-193315130 + E=3e-132
TGCCAGTATTTTATCAAGGGGGAGATTTGTAGAAGATTAGTAAGGTCAGAGAAGTTTAATTTTTGAATTAATTGTATCAAGTGTATGGC

ATTAAGATAATGGTTGTTTGCAACTTAACTTTGGAAGGGGATCATCATTTATTAGTCCTTTTTGGTATTGAAATTTTCTATTTTTTATA

AAAGCATTATCATATA|TATTTTCACATAGTTGAGAATGTTTCCATATGTCTGGCATGTTTGTAATCCAGCACTTTGAGAGGTGGAAGC
                  >chr9:69073457-69073670 - E=5e-95
AGGAGAATCACTTAAGCCCAGGAGTTCAAAATCAGCCTAGGCAACAAAAGGAGACCCCCATCTCTACAAAAATATTAAGAAATTAGCTG

GGTATGGTGGTACAAGCCTGTGGTCCCAGCTACTCGGGAGTCTGAGGTGAGA
```
